# Supplementary material for: Family planning in Pacific Island Countries and Territories (PICTs): A scoping review
Source: PLoS One. 2021 Aug 5;16(8):e0255080. doi: 10.1371/journal.pone.0255080 (PMC8341522; doi:10.1371/journal.pone.0255080)
Supplement: S1 Appendix — (PDF) [file pone.0255080.s001.pdf]

S1 Appendix. Review Protocol

Title:

Family planning in Pacific Island Countries and Territories (PICTs): A scoping review

Background

In order to improve the provision of family planning services in PICTs context, there is an urgent need to gather information to ensure appropriate family planning services reach everyone regardless of who they are and where they live. The available literature tends to report on barriers and enablers to access family planning services and limited information is available on what is happening at the service provision level in PICTs. To date no study has compiled the literature on family planning service provision in PICTs. This review will explore existing evidence to providing family planning services in the PICTs and assess their suitability for the Pacific context. A scoping review approach will be used as topic is broad and little is known, about the topic. Relevant materials in both the grey and published literature will be searched. The Arksey and O'Malley (Arksey & O'Malley, 2005) methodological framework below will be used and enhancement suggested by Levac, Colquhoun and Obrien (Levac, Colquhoun, & O'Brien, 2010).

Aim

To explore existing evidence about family planning service provision in PICTs, and identify successes and challenges to inform improved family planning services.

Research questions

1. How have family planning services been implemented in Pacific Island Countries and Territories (PICTs) from 1994 to current?
2. What are the successes and challenges in providing family planning services in PICTs?

Methodology

Design

A scoping review design has been chosen as there is limited published about family planning service provision in PICTs. This review will help establish and map key concepts from a wide range of literature, and identify gaps to inform further research. The review will examine and extract the evidence using the Arksey and O'Malley methodological framework of scoping review (Arksey & O'Malley, 2005), along with suggestions from Levac, Colquhoun and O'brien (Levac et al., 2010). The Joanna Briggs Institute's approach (Peters et al., 2015) to conducting scoping reviews will also (Tricco et al., 2018)be integrated in this method.

Study setting

This scoping review will consider all applicable publications on family planning or contraceptive services conducted in the PICTs. As there are a limited number of peer-reviewed publications available, the search will not be restricted to published peer-reviewed articles but include unpublished work (grey literature). This review will focus on family planning services provided to everyone (women, men and young people) in the PICTs context.

Sampling strategy

Relevant articles for this review will be extracted from searches using the four stages Preferred Reporting Items for Systematic Reviews and Meta-Analysis extension for Scoping Reviews (PRISMA-ScR) flow diagram (Tricco et al., 2018).

1. Identification – articles will be identified through database searching and manual searching, duplicates removed.
2. Screening - titles and abstracts will be screened according to the inclusion criteria
3. Eligibility/selection – full-text articles will be assessed for eligibility, those not meeting inclusion criteria will be excluded
4. Inclusion – remaining articles will be included

Inclusion criteria

1. Any article or publication that meets the following criteria will be included for full-text screening:
    a. focuses on family planning or contraceptive services
    b. sexual and reproductive health services that includes the family planning component
    c. reporting successes and challenges in providing family planning service
2. The article or publication is written in English
3. The article or publication was published from January 1994 to current

The inclusive date was selected to reflect the progress from the 1994 International Conference on Population and development (ICPD) in Cairo when commitments to reproductive health rights and voluntary family planning was established as a fundamental human right and also encompassing the period of the millennium development goals (MDGs) and beginning of the sustainable development goals (SDGs).

For the purpose of this review, the World Health Organisation's (WHO) definition of family planning is used:

"Family planning allows individuals and couples to anticipate and attain their desired number of children and spacing and timing of their births. It is achieved through use of contraceptive methods and the treatment of involuntary infertility" (World Health Organisation, 2010). Family planning is part of a broader sexual and reproductive health definition used by WHO that promotes the rights of women and men to make informed decisions and have access to safe, effective, affordable and acceptable method of family planning.

Data collection/search strategy for identification of studies

Electronic databases searching:

Family planning research is found in the disciplines of medicine, public health, education and nursing.

i. An exploratory search will be carried out in Scopus and references downloaded.

ii. A comprehensive search will be conducted in:

a) MEDLINE/OVID

b) CINAHL

c) Google Scholar

Manual searching:

d) Grey literature will be searched from the following international organisations: WHO, UNFPA, UNICEF, PSRH, SPC for reports related to assessment of family planning services in the Pacific Island Countries and Territories.

e) Bibliographic references will be hand searched for relevant information.

All relevant references will be exported to Endnote and downloaded.

PCC table

**P**opulation	men<br>women<br>Contraceptive users<br>Family planning service users<br>Family planning service providers<br>Reproductive health service providers<br>Men as partners in reproductive health<br>Adolescen*
**Concept**	Family planning program, pregnancy<br>Contraceptive methods<br>Contracepti*<br>Family planning service/reproductive health service
**Context**	Pacific Island Countries and Territories<br>Pacific<br>Pacific Island Countries<br>Melanesia<br>Polynesia<br>Micronesia<br>American Samoa, Cook Islands, Federated States of Micronesia, Fiji, French Polynesia, Guam, Kiribati, Marshall Islands, Nauru, New Caledonia, Niue, Northern Mariana Islands, Palau, Papua New Guinea, Pitcairn Islands, Samoa, Solomon Islands, Tokelau, Tonga, Tuvalu, Vanuatu, Wallis and Futuna.

Search Terms:

1. “family planning services” OR “family planning approaches” OR “reproductive health services” OR “sexual health” OR “sexual reproductive health services” OR “sexual and reproductive health services” OR adolescent sexual and reproductive health OR contracepti* OR “health service provision” OR “family planning program” OR “pregnan*

AND

2. barrier* OR challenge* OR opportunit* OR enabler* OR success* OR service* OR program*

AND

3. wom?n OR m?n OR youth* OR ‘young people” OR “young adult” OR “women of childbearing age” OR “women of reproductive age” OR “male involvement in reproductive health” OR “family planning service provider” OR “family planning service user” OR “health service provider” OR “reproductive health service provider”.

AND

4. health OR “public health” OR health service delivery OR primary health care

5. “pacific island countries and territories” OR pacific OR “pacific island countries” OR “pacific region” OR “oceania countries” OR “asia pacific countries” OR “western pacific region” OR “low and middle income countries” OR melanesia OR polynesia OR micronesia OR “american samoa”, “cook Islands” OR “federated states of micronesia” OR fiji OR “french polynesia” OR guam OR kiribati OR “marshall islands” OR nauru OR “new caledonia” OR niue OR “northern mariana islands” OR palau OR “papua new guinea” OR samoa OR “solomon islands” OR tokelau OR tonga OR tuvalu OR vanuatu OR “wallis and futuna”.

Grey literature search terms:

1. “family planning services” OR “family planning approaches” OR “reproductive health services” OR “sexual health” OR “sexual reproductive health services” OR “sexual and reproductive health services” OR adolescent sexual and reproductive health OR contracepti* OR “health service provision” OR “family planning program” OR “pregnan*

AND

2. barrier* OR challenge* OR opportunit* OR enabler* OR success*

AND

3. wom?n* OR m?n OR youth* OR ‘young people” OR “young adult” OR “women of childbearing age” OR “women of reproductive age” OR “male involvement in reproductive health” OR “family planning service provider” OR “family planning service user” OR “health service provider” OR “reproductive health service provider”.

AND

4. health OR “public health” OR “health service delivery” OR “primary health care”

AND

5. “pacific island countries and territories” OR pacific OR “pacific island countries” OR “pacific region” OR “oceania countries” OR “asia pacific countries” OR “western pacific region” OR “low and middle income countries” OR melanesia OR polynesia OR micronesia OR “american samoa”, “cook Islands” OR “federated states of micronesia” OR fiji OR “french polynesia” OR guam OR kiribati OR “marshall islands” OR nauru OR “new caledonia” OR niue OR “northern mariana islands” OR palau OR “papua new guinea” OR samoa OR “solomon islands” OR tokelau OR tonga OR tuvalu OR vanuatu OR “wallis and futuna”.

Quality assessment

A Quality assessment of included articles will not be performed as this scoping review aimed to map existing literature relating to family planning service provision.

Planned Analysis

The included articles will be charted in an Excel spreadsheet to organise important findings. Data will be collated, interpreted, summarised and thematically analysed. Findings will be presented using a narrative style.

Ethical considerations

A scoping review does not require ethical approval as it examines data from existing literature that is available. However, given the sensitivity of this area of research, authors will need to handle the information with confidentiality and sensitivity.

Dissemination

This review will be published in an appropriate journal.

References

Arksey, H., & O'Malley, L. (2005). Scoping studies: towards a methodological framework. *International Journal of Social Research Methodology, 8*(1), 19-32. doi:10.1080/1364557032000119616

Levac, D., Colquhoun, H., & O'Brien, K. K. (2010). Scoping studies: advancing the methodology. *Implementation science : IS, 5*(1), 69-69. doi:10.1186/1748-5908-5-69

Peters, M. D. J., Godfrey, C. M., Khalil, H., McInerney, P., Parker, D., & Soares, C. B. (2015). Guidance for conducting systematic scoping reviews. *JBI Evidence Implementation, 13*(3), 141-146. doi:10.1097/xeb.0000000000000050

Tricco, A. C., Lillie, E., Zarin, W., O'Brien, K. K., Colquhoun, H., Levac, D., . . . Weeks, L. (2018). PRISMA extension for scoping reviews (PRISMA-ScR): checklist and explanation. *Annals of internal medicine, 169*(7), 467-473.

World Health Organisation. (2010). *The ABC's of Family Planning*. Retrieved from Canada: https://www.who.int/reproductivehealth/topics/family_planning/en/
