# Supplementary material for: Family planning in Pacific Island Countries and Territories (PICTs): A scoping review
Source: PLoS One. 2021 Aug 5;16(8):e0255080. doi: 10.1371/journal.pone.0255080 (PMC8341522; doi:10.1371/journal.pone.0255080)
Supplement: S3 Appendix — (PDF) [file pone.0255080.s003.pdf]

S3 Appendix. Eligibility/Inclusion criteria

1. Published from January 1994 to current – to reflect the progress from the 1994 International Conference on Population and development (ICPD) in Cairo when commitments to reproductive health rights and voluntary family planning was established as a fundamental human right and also encompassing the period of the millennium development goals (MDGs) and beginning of the sustainable development goals (SDGs).
2. Published in English
3. Peer-reviewed and grey literature
4. Any publication that focuses on family planning approaches in PICTs
5. Articles that included links to family planning related facilities and Pacific contexts.
