# Supplementary material for: Family planning in Pacific Island Countries and Territories (PICTs): A scoping review
Source: PLoS One. 2021 Aug 5;16(8):e0255080. doi: 10.1371/journal.pone.0255080 (PMC8341522; doi:10.1371/journal.pone.0255080)
Supplement: S4 Appendix — (PDF) [file pone.0255080.s004.pdf]

S4 Appendix. Key words

1) Family planning services	
Key words	“family planning services” OR “family planning approaches” OR “reproductive health services” OR “sexual health” OR “sexual reproductive health services” OR “sexual and reproductive health services” OR adolescent sexual and reproductive health OR “maternal health services” OR contracepti* OR “health service provision” OR “family planning program” OR “postnatal services” “pregnan*
**AND**	
**2. Challenges or Successes**	
Key words	challenge* OR success* OR opportunit* OR enabler* OR barrier* OR service* OR program*
**AND**	
**3. Population**	
Key words	wom?n OR “male involvement in reproductive health” OR “family planning service provider” OR “family planning service user” OR “health service provider” OR “reproductive health service provider”.
**AND**	
**4. Context**	
Key words	“pacific island countries and territories” OR pacific OR “pacific island countries” OR “pacific region” OR “oceania countries” OR “asia pacific countries” OR “western pacific region” OR “low and middle income countries” OR melanesia OR polynesia OR micronesia OR “american samoa”, “cook Islands” OR “federated states of micronesia” OR fiji OR “french polynesia” OR guam OR kiribati OR “marshall islands” OR nauru OR “new caledonia” OR niue OR “northern mariana islands” OR palau OR “papua new guinea” OR samoa OR “solomon islands” OR tokelau OR tonga OR tuvalu OR vanuatu OR “wallis and futuna”.

Limits: English language, Publication Year (1994 – 2019)
