# Supplementary material for: Family planning in Pacific Island Countries and Territories (PICTs): A scoping review
Source: PLoS One. 2021 Aug 5;16(8):e0255080. doi: 10.1371/journal.pone.0255080 (PMC8341522; doi:10.1371/journal.pone.0255080)
Supplement: S5 Appendix — (PDF) [file pone.0255080.s005.pdf]

S5 Appendix: Medline Ovid: Search Strategy

#	Query	Results
1	Family Planning Services/	25,142
2	Contraception/	20,183
3	reproductive health/ or sexual health/ or women's health/ or maternal health/	34,036
4	Adolescent health.mp. or Adolescent Health/	12,060
5	1 or 2 or 3 or 4	80,290
6	barrier*.mp.	335,065
7	challeng*.mp.	903,041
8	fail*.mp.	1,313,369
9	opportunity*.mp.	133,014
10	enabler*.mp.	3,819
11	success*.mp.	1,205,454
12	6 or 7 or 8 or 9 or 10 or 11	3,529,494
13	women.mp. or Women/	1,035,068
14	Female/	8,990,221
15	men.mp. or Men/	536,462
16	Male/	8,841,918
17	adolescent health/ or family health/ or men's health/ or public health/ or reproductive health/ or sexual health/	116,152
18	13 or 14 or 15 or 16 or 17	12,090,822
19	pacific islands/ or melanesia/ or fiji/ or new caledonia/ or papua new guinea/ or vanuatu/ or micronesia/ or guam/ or palau/ or polynesia/ or pitcairn island/ or samoa/ or tonga/	14,387
20	(pacific island countries and territories).mp. [mp=title, abstract, original title, name of substance word, subject heading word, floating sub-heading word, keyword heading word, organism supplementary concept word, protocol supplementary concept word, rare disease supplementary concept word, unique identifier, synonyms]	105
21	micronesia.mp. or exp Micronesia/	2,480
22	melanesia.mp. or exp Melanesia/	6,848
23	polynesia.mp. or exp Polynesia/	11,377
24	pacific islands.mp. or Pacific Islands/	4,967
25	"american samoa".mp. or American Samoa/	409
26	"cook islands".mp.	228
27	"federated states of micronesia".mp.	266
28	fiji.mp.	2,122

29	"french polynesia".mp.	1,040
30	guam.mp.	1,377
31	kiribati.mp.	214
32	"marshall islands".mp.	312
33	nauru.mp.	163
34	"new caledonia".mp.	1,673
35	niue.mp.	77
36	"northern mariana islands".mp.	140
37	palau.mp.	443
38	"papua new guinea".mp.	5,514
39	"pitcairn islands".mp. or Pitcairn Island/	17
40	samoa.mp.	1,211
41	"solomon islands".mp.	864
42	tokelau.mp.	97
43	tonga.mp.	527
44	tuvalu.mp.	77
45	vanuatu.mp.	755
46	(wallis and futuna).mp. [mp=title, abstract, original title, name of substance word, subject heading word, floating sub-heading word, keyword heading word, organism supplementary concept word, protocol supplementary concept word, rare disease supplementary concept word, unique identifier, synonyms]	43
47	19 or 20 or 21 or 22 or 23 or 24 or 25 or 26 or 27 or 28 or 29 or 30 or 31 or 32 or 33 or 34 or 35 or 36 or 37 or 38 or 39 or 40 or 41 or 42 or 43 or 44 or 45 or 46	28,550
48	5 and 12 and 18 and 47	77
49	limit 48 to (english language and humans and yr="1994 - 2019")	43
