# Supplementary material for: Family planning in Pacific Island Countries and Territories (PICTs): A scoping review
Source: PLoS One. 2021 Aug 5;16(8):e0255080. doi: 10.1371/journal.pone.0255080 (PMC8341522; doi:10.1371/journal.pone.0255080)
Supplement: S6 Appendix — (PDF) [file pone.0255080.s006.pdf]

S6 Appendix. Selection of sources of evidence

Background information:<br>Paper identification<br>Does the title fit?
Screening questions and assessments<br>1. Is the study published from 1994 onwards? (Yes/No/Unclear)<br>2. Is the study report published in English? (Yes/No/Unclear)<br>3. Is the study report conducted in the Pacific Island Countries and Territories? (Yes/No/Unclear)<br>4. Does the publication describe reproductive health including family planning services or programs? (Yes/No/Unclear)<br>5. Does the publication describe the challenges and successes to providing family planning services in the Pacific? (Yes/No/Unclear)
Assessment (INCLUDE/EXCLUDE/MAYBE)<br>Included (If answers yes to questions 1, 2, 3 and yes to either 4 or 5)<br>Excluded following initial screening (Title and Abstract)<br>Excluded following full text screening<br>If 'excluded', give reason why or do we need to reference check for other potential studies)<br>If 'maybe', comment and have another author to review followed by team discussion before decision is made to include or exclude
