# Supplementary material for: Family planning in Pacific Island Countries and Territories (PICTs): A scoping review
Source: PLoS One. 2021 Aug 5;16(8):e0255080. doi: 10.1371/journal.pone.0255080 (PMC8341522; doi:10.1371/journal.pone.0255080)
Supplement: S7 Appendix — (PDF) [file pone.0255080.s007.pdf]

S7 Appendix. Data charting process

Background information<br>Paper identification number<br>Publication title<br>Type of publication<br>Year of publication<br>Authors names<br>Journal name<br>Country/Location
Study design and analysis<br>Study type<br>Aim/focus of study<br>Sample size/age range of participants/gender<br>Data collection methods (e.g. focus group discussions/ interviews/surveys)<br>Data analysis<br>Key findings
Family planning service provision approaches<br>How family planning service is provided
Challenges/Successes/Enabling environments<br>Important details on the challenges and successes of family planning services<br>Potential enabling environments to improve service provision
