# Supplementary material for: Family planning in Pacific Island Countries and Territories (PICTs): A scoping review
Source: PLoS One. 2021 Aug 5;16(8):e0255080. doi: 10.1371/journal.pone.0255080 (PMC8341522; doi:10.1371/journal.pone.0255080)
Supplement: S8 Appendix — (PDF) [file pone.0255080.s008.pdf]

S8 Appendix. Examples of coding process

Meaning Unit	Code	Theme
‘Family planning services focus on married couples, not available to the unmarried regardless of age’	Availability of service	Family planning service in the Pacific
‘Higher education level of users and sexual reproductive health education are not enough to reduce barriers to access’	Education level<br>Knowledge	Education, knowledge and attitudes
