# Supplementary material for: Family planning in Pacific Island Countries and Territories (PICTs): A scoping review
Source: PLoS One. 2021 Aug 5;16(8):e0255080. doi: 10.1371/journal.pone.0255080 (PMC8341522; doi:10.1371/journal.pone.0255080)
Supplement: Attachment — Submitted filename: FFP in PICTs A scoping review - Reviewer Comments KimR.docx [file pone.0255080.s010.docx]

Page 5: “The population growth rate of some countries are still too high to ensure viable, sustainable futures and the slow rate of economic development has not kept pace with population growth. … ”

*Perhaps there could be some value add in a sentence summarising summary of government budget / donor funding or investment in the health sector to provide some kind of ‘link’ for this and the following sentences in the paragraph or to strengthen the argument about the limited ‘supply side’ and universal access in relation to SRH?*

Page 6: “The CPR indicates the percentage of women of reproductive age (15-49 years) using any contraceptive method at a given point in time.”

While this is the technically correct definition, it is typically operationalised in household surveys as women who are currently married or in union who are using FP or their partner is – see for example MICS definition at <https://mics.unicef.org/tools>. Suggest adding some more contextual details to the definition in terms of how it is collected and published in surveys like DHS, MICS etc. Perhaps the discussion around FP users could have been enriched through the Ministry of Health annual reports (grey literature) that usually contain information from administrative databases (health information systems) about users of government FP services and products etc – but understandably this source would probably not have been part of the search results, and would have been included in some of the work referenced/cited. And its likely that this grey literature would not meet all of the eligibility criteria.

DHS survey CPR data presented in Table 1: Page 11+. Authors should be consistent in referring to results of CPR from DHS surveys. Kiribati is correctly sited as “Contraceptive prevalence rate – 22% (married women), 16.5% (all women)”; whereas DHS survey rates for Marshall Is, Nauru, PNG, Samoa, Solomon Is, Vanuatu all have “Contraceptive prevalence rate – xx%” and it seems that this is currently married women aged 15-49.

Page 26: “Evidence about the extent to which FP services are reaching those who use the service and how the service was provided at the health facility level is lacking in the literature [30, 46].” I wonder if this sentence could be expanded to include reference to reasons for low uptake where services are available and the socio-cultural barriers identified in the research?

Page 27: “The availability of quality and relevant SRH, including FP data was a common and ongoing challenge in PICTs. Robertson [35] and UNFPA [14, 68, 70, 72] documented poor reporting systems and processes that made it difficult to determine whether valid data existed for most countries.” and following paragraph. Perhaps a qualifying sentence or text could be added to provide perspective for the need for DHS and similar ‘prevalence’ surveys because the scope and coverage of MoH management information systems and associated administrative data are inadequate to derive quality CPRs?

Discussion – much improved.

Conclusion – key messages are captured well. Page 38: “Family planning services in PICTs do not reach all age groups, genders and unmarried young people.” Could add reference to all locations, religions, abilities – reference to different ethnic groups, persons living with disability? “Many contextual challenges remain in each Pacific country and territory.” [in terms of both supply and demand side for FP/SRH]
